# A union of two rare pathologies: small bowel diverticula perforation secondary to impacted gallstone ileus

**DOI:** 10.1093/jscr/rjaf026

**Published:** 2025-01-26

**Authors:** Joseph Do Woong Choi, Pranesh de Silva, Craig Lynch, Stephen Pillinger, Praveen Ravindran

**Affiliations:** Discipline of Surgery, Faculty of Medicine and Health, The University of Sydney, Parramatta Road, Sydney, New South Wales 2006, Australia; Department of Colorectal Surgery, Sydney Adventist Hospital, 185 Fox Valley Road, Wahroonga, Sydney, New South Wales 2076, Australia; Department of Colorectal Surgery, Sydney Adventist Hospital, 185 Fox Valley Road, Wahroonga, Sydney, New South Wales 2076, Australia; College of Health and Medicine, Australian National University, 54 Mills Road, Acton, Australian Capital Territory 2601, Australia; Department of Colorectal Surgery, Sydney Adventist Hospital, 185 Fox Valley Road, Wahroonga, Sydney, New South Wales 2076, Australia; College of Health and Medicine, Australian National University, 54 Mills Road, Acton, Australian Capital Territory 2601, Australia; Department of Colorectal Surgery, Sydney Adventist Hospital, 185 Fox Valley Road, Wahroonga, Sydney, New South Wales 2076, Australia; College of Health and Medicine, Australian National University, 54 Mills Road, Acton, Australian Capital Territory 2601, Australia; Department of Colorectal Surgery, Sydney Adventist Hospital, 185 Fox Valley Road, Wahroonga, Sydney, New South Wales 2076, Australia; College of Health and Medicine, Australian National University, 54 Mills Road, Acton, Australian Capital Territory 2601, Australia

**Keywords:** gallstone ileus, jejunal diverticulosis, small bowel obstruction, perforated small bowel diverticulitis

## Abstract

An 84-year-old lady presented with 1 day history of sudden onset generalized abdominal pain, fevers, and peritonism. Computed tomography was suggestive of a mid-small bowel perforation associated with a distal ovoid soft tissue density structure without pneumobilia. An urgent laparotomy demonstrated two areas of jejunal diverticula necrosis and perforation associated with a 3 cm luminal mass in the proximal ileum, and proximal small bowel dilatation. A 100 cm small bowel resection incorporating the mass and perforated jejunal diverticula and primary stapled anastomosis were performed. Histopathology surprisingly demonstrated cholelithiasis consistent with a gallstone ileus and necrotic, perforated jejunal diverticulitis. The patient had no recurrent symptoms at 6 weeks follow-up. The authors report an uncommon and unexpected occurrence of small bowel diverticulitis perforation as a rare complication of gallstone ileus.

## Introduction

Gallstone ileus is a type of mechanical intestinal obstruction involving luminal gallstone impaction [1]. It is an uncommon yet serious complication of cholelithiasis, occurring in 0.15%–1.5% of cases with a mortality rate up to 22.7% [1, 2]. Perforation of the small intestine due to obstructing gallstone is a rare complication of gallstone ileus with <10 cases having been reported [3]. The authors present a unique case of gallstone ileus with associated perforated jejunal diverticulitis secondary to high proximal intraluminal pressure.

## Case report

**Figure 1:**
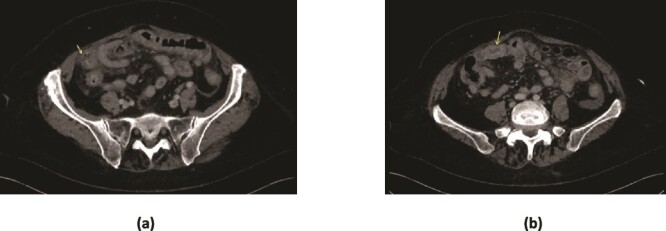
CT scan demonstrating (a) marked mural thickening of the small bowel in the right lower quadrant with areas of mural discontinuity (arrow) and peritoneal free fluid suggestive of perforation (b) ovoid soft tissue density in a small bowel loop, with collapsed small bowel loops distally.

**Figure 2:**
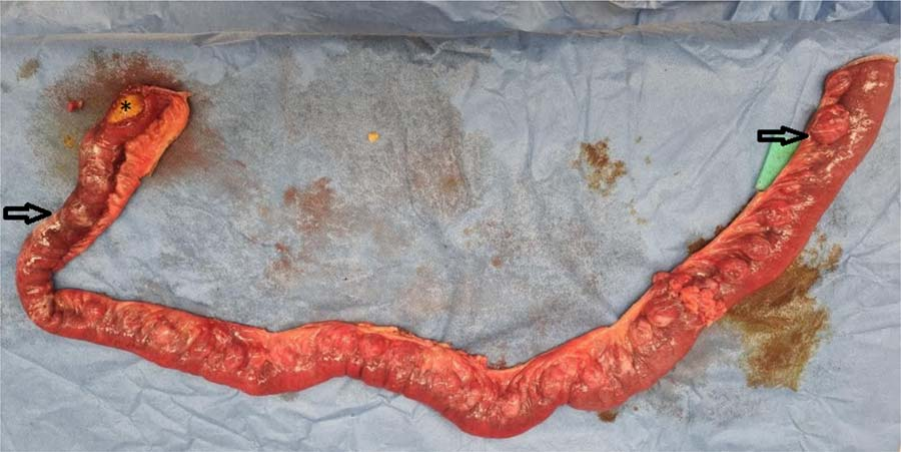
Intraoperative photo of 100 cm resected mid small bowel demonstrating multiple small bowel diverticula with two areas of perforation related to it (arrows) and a gallstone distal to the perforation (star).

An 84-year-old lady presented with 1 day history of sudden onset severe generalized abdominal pain radiating to the back. This was associated with four vomits, and she had passed a small bowel motion earlier in the day. Her background history is significant for type 2 diabetes mellitus controlled with metformin. She did not have any previous abdominal surgeries, or gallstones. There was no family history of gastrointestinal diverticula, gallstones or malignancies. She resides at home, being the primary carer for her husband. On examination, she was febrile up to 38.2°C, blood pressure 150/69, heart rate of 90/minute, respiratory rate 18/minute and saturating at 95% on room air. Her abdomen was mildly distended, and generally tender with focal peritonism in the central abdomen. Her white cell count (WCC) was elevated at 12.0 × 10^9^/L (reference: 4.0–11.0 × 10^9^/L), neutrophils 10.50 × 10^9^/L (reference: 2.0–7.50 × 10^9^/L) and c reactive protein (CRP) of 40.0 (reference: 0.0–5.0). A computed tomography (CT) scan demonstrated marked mural thickening of the small bowel in the right lower abdomen with two areas of mural discontinuity suggestive of small bowel perforation. There was moderate ascites, without extraluminal gas or pneumatosis intestinalis. Distally, there was a non-specific ovoid soft tissue density structure in a small bowel loop, beyond which small bowel loops were collapsed (Fig. 1). There was intrahepatic (up to 7 mm) and extrahepatic (13 mm) biliary tree dilatation without pneumobilia. The gallbladder had fundal mural thickening, without features of cholecystitis. There was no CT evidence of a biliary-enteric fistula. She underwent an urgent laparotomy which demonstrated four quadrant peritonitis, and two separate areas of necrotic mid small bowel, jejunal diverticula with perforations at the mesenteric border. There was a palpable 3 cm solid intraluminal mass distal to the perforations in the proximal ileum, significant proximal small bowel dilatation and multiple other uncomplicated jejunal diverticula (Fig. 2). There were no other luminal masses during a small bowel run. One hundred centimetres of small bowel was resected, and a side-to-side functional end to end small bowel anastomosis was performed 30 cm distal to the duodenojejunal flexure to 120 cm proximal from the ileo-caecal valve using a GIA 80 mm blue reload stapler. Histopathology demonstrated perforated necrotic small bowel diverticulitis without malignancy, with the palpable intraluminal mass confirmed as cholelithiasis. The patient had an uncomplicated recovery but due to deconditioning, was referred for inpatient rehabilitation after two weeks. At her 6 weeks follow-up, she reported no recurrent symptoms.

## Discussion and conclusions

The authors were unaware prior to the laparotomy that the patient had jejunal perforation from pressure effects secondary to gallstone ileus as there was no history of gallstones, and no features of pneumobilia or a biliary-enteric fistula on CT. The authors initially suspected that the ovoid soft tissue density on imaging was representative of a small bowel neoplasm. Symptoms and clinical signs of gallstone ileus are usually non-specific including abdominal pain at presentation in 91.5% of patients, abdominal distension and vomiting in 84.7% and 59.7% of all patients, respectively [4]. The most common impaction site of the gallstone are the ileum (50%–60.5%), jejunum (16.1%–26.9%), duodenum (3.5%–14.6%), and colon (3%–4.1%) [1].

CT has a high sensitivity (93%) and specificity (100%) in diagnosing gallstone ileus [5]. In the presented case, the CT highlighted marked mural thickening of small bowel and an ovoid structure with collapsed small bowel loops distally without evidence of pneumobilia (Fig. 1). The classic radiologic sign of gallstone ileus ‘Rigler triad’ includes presence of pneumobilia, intestinal obstruction and an ectopic gallstone [2]. In hindsight, our patient had two out of three of Rigler triad. Perforation secondary to gallstone ileus is very rare, with a review of 458 cases of gallstone ileus reporting only two cases of jejunal perforation [8]. Perforation often occured at the site of impaction, or previous sites of obstruction due to pressure necrosis of the jejunal wall from the gallstone [6, 7].

Additionally, small bowel diverticula is an infrequent occurrence with a reported prevalence at autopsy of 0.3%–1.3% [9]. They are often observed in patients aged 60–70 years [10]. Its aetiology remains unknown, but believed to result from abnormalities in intestinal peristalsis, intestinal dyskinesis and high intraluminal pressure [11]. They emerge only at the small bowel mesenteric border where the mesenteric vessels penetrate into the muscular layer of the small intestine [11]. They are often found incidentally on imaging or intraoperatively, and the complications are much more uncommon, including acute diverticulitis, bleeding, perforation, stricturing, small bowel obstruction and fistulation [12].

A review of existing literature highlighted that there have been eight reported cases of jejunal diverticulitis with simultaneous enterolith obstruction [12]. In our case, the patient had necrotic jejunal diverticula perforations secondary to high proximal intraluminal pressure effects from concomitant impacted gallstone ileus.

There have only been three other reported cases of gallstone ileus complicated by jejunal diverticula perforation [6, 8]. In these cases, they reported the presence of pneumobilia, contrary to our study which had no such finding. The surgical management was similar in that resection of the perforated small bowel segment, stone extraction and primary small bowel anastomosis was performed. Controversies remain on the most appropriate approach and extent of surgery for gallstone ileus, particularly in regards to addressing the biliary-enteric fistula [8]. The first approach is enterolithotomy +/− small bowel resection alone. Although this is associated with lower morbidity and mortality compared to one or two-stage procedures, its main drawback is unaddressed fistula, with a reported recurrence up to 62.6% of patients within 6 weeks [13]. One-stage procedure of enterolithotomy, cholecystectomy, and fistula closure is usually performed in highly select patients who present with acute cholecystitis, gangrenous gallbladder, or residual gallstones [8]. Finally, a two-stage procedure consisting of initial enterolithotomy, followed by cholecystectomy and closure of biliary-enteric fistula tract after 4–6 weeks has also been described [7]. In our patient, the authors chose not to plan for further surgery to address a possible biliary-enteric fistula due to advanced age, deconditioning and frailty.

## Limitations

The case study is not without limitations. First, it highlighted successful management of one patient who unusually had two rare pathologies that acted synergistically. Thus, the treatment pathway described was not based on extensive evidence, as they do not exist in the literature. Additionally, the authors chose not to further investigate or plan for further surgery to address the possible biliary-enteric fistula, however, this may not be generalizable as younger patients with less comorbidities may benefit from a two-stage procedure to minimize the lifetime risk of recurrent gallstone ileus. Despite these limitations we have highlighted an unusual life-threatening presentation requiring prompt surgical management, and recommend general surgeons to maintain a broad set of differentials when faced with patients presenting with acute abdominal pain.

## Data Availability

Available after obtaining permission from the corresponding author.
